# Geographical and temporal distribution of SARS-CoV-2 clades in the WHO European Region, January to June 2020

**DOI:** 10.2807/1560-7917.ES.2020.25.32.2001410

**Published:** 2020-08-13

**Authors:** Erik Alm, Eeva K Broberg, Thomas Connor, Emma B Hodcroft, Andrey B Komissarov, Sebastian Maurer-Stroh, Angeliki Melidou, Richard A Neher, Áine O’Toole, Dmitriy Pereyaslov, The WHO European Region sequencing laboratories and GISAID EpiCoV group, The WHO European Region sequencing laboratories and GISAID EpiCoV group, The WHO European Region sequencing laboratories and GISAID EpiCoV group, Niko Beerenwinkel, Susana Posada-Céspedes, Kim Philipp Jablonski, Pedro Falé Ferreira, Ivan Topolsky, Tatjana Avšič-Županc, Miša Korva, Mario Poljak, Samo Zakotnik, Tomaž Mark Zorec, Karoline Bragstad, Olav Hungnes, Kathrine Stene-Johansen, Chantal Reusken, Adam Meijer, Harry Vennema, Lidia Ruiz-Roldán, María Alma Bracho, Neris García-González, Álvaro Chiner-Oms, Irving Cancino-Muñoz, Iñaki Comas, Galo A Goig, Manuela Torres-Puente, Mariana G López, Llúcia Martínez-Priego, Giuseppe D'Auria, Loreto Ferrús-Abad, Griselda de Marco, Inmaculada Galan-Vendrell, Sandra Carbó-Ramirez, Paula Ruíz-Hueso, Mireia Coscollá, Katerina Polackova, Lenka Kramna, Ondrej Cinek, Jan Richter, George Krashias, Christina Tryfonos, Stavros Bashiardes, Dana Koptides, Christina Christodoulou, Barbara Bartolini, Cesare Em Gruber, Antonino Di Caro, Concetta Castilletti, Fabrizio Stefani, Sara Giordana Rimoldi, Francesca Romeri, Franco Salerno, Stefano Polesello, Alexander Nagy, Helena Jirincova, Jaromira Vecerova, Ludmila Novakova, Samuel Cordey, Marine Murtskhvaladze, Nato Kotaria, Tobias Schär, Christian Beisel, Oliver Vugrek, Filip Rokić, Lovro Trgovec-Greif, Igor Jurak, Tomislav Rukavina, Neven Sučić, Kristian Schønning, Søren M Karst, Rasmus H Kirkegaard, Thomas Y Michaelsen, Emil Aa Sørensen, Simon Knutson, Jakob Brandt, Vang Le-Quy, Trine Sørensen, Celine Petersen, Martin Schou Pedersen, Sanne Løkkegaard Larsen, Marianne Nielsine Skov, Morten Rasmussen, Jannik Fonager, Anders Fomsgaard, Rinat Amirovich Maksyutov, Elena Vasil'Evna Gavrilova, Oleg Victorovich Pyankov, Sergey Alexandrovich Bodnev, Tatyana Vladimirovna Tregubchak, Alexander Nikolayevich Shvalov, Denis Victorovich Antonets, Paola Cristina Resende, Stephanie Goya, Amandine Perrin, Raphael Tc Lee, Shilpa Yadahalli, Alvin X Han, Colin A Russell, Stefan Schmutz, Maryam Zaheri, Verena Kufner, Michael Huber, Alexandra Trkola, Markus Antwerpen, Mathias C Walter, Sylvie van der Werf, Fabiana Gambaro, Sylvie Behillil, Vincent Enouf, Flora Donati, Monta Ustinova, Vita Rovite, Janis Klovins, Oksana Savicka, Anke K Wienecke-Baldacchino, Catherine Ragimbeau, Guillaume Fournier, Joël Mossong, Stephan W Aberle, Mattias Haukland, Theresa Enkirch, Abdolreza Advani, Maria Lind Karlberg, Oskar Karlsson Lindsjö, Sandra Broddesson, Monika Sláviková, Martina Ličková, Boris Klempa, Edita Staroňová, Elena Tichá, Tomáš Szemes, Diana Rusňáková, Tanja Stadler, Josep Quer, Andres Anton, Cristina Andres, Maria Piñana, Damir Garcia-Cehic, Tomas Pumarola, Jacques Izopet, Georgia Gioula, Maria Exindari, Anna Papa, Dimitrios Chatzidimitriou, Symeon Metallidis, Stella Pappa, Milan Macek Jr, Jan Geryk, Petr Brož, Aleš Briksí, Petr Hubáček, Pavel Dřevínek, Miroslav Zajac, Petr Kvapil, Michal Holub, Kateřina Kvapilová, Adam Novotný, Martin Kašný, Petr Klempt, Olli Vapalahti, Teemu Smura, Tarja Sironen, Philippe Selhorst, Colin Anthony, Kevin Ariën, Etienne Simon-Loriere, Lukasz Rabalski, Krystyna Bienkowska-Szewczyk, Vítor Borges, Joana Isidro, João Paulo Gomes, Raquel Guiomar, Pedro Pechirra, Inês Costa, Sílvia Duarte, Luís Vieira, Krzysztof Pyrc, Neta S Zuckerman, Shahlo Turdikulova, Alisher Abdullaev, Dilbar Dalimova, Abror Abdurakhimov, Adriano Tagliabracci, Federica Alessandrini, Filomena Melchionda, Valerio Onofri, Chiara Turchi, Patrizia Bagnarelli, Stefano Menzo, Sara Caucci, Laura Di Sante, Alexandra Popa, Jakob-Wendelin Genger, Benedikt Agerer, Alexander Lercher, Lukas Endler, Mark Smyth, Thomas Penz, Michael Schuster, Martin Senekowitsch, Jan Laine, Christoph Bock, Andreas Bergthaler, Alexandr Shevtsov, Ruslan Kalendar, Yerlan Ramanculov, Alexander Graf, Maximilian Muenchhoff, Oliver T Keppler, Stefan Krebs, Helmut Blum, Alessandro Marcello, Danilo Licastro, Pierlanfranco D'Agaro, Florian Laubscher, Dejan Vidanovic, Bojana Tesovic, Jeremy Volkening, Nicola Clementi, Nicasio Mancini, Maja Rupnik, Aleksander Mahnic, Andreas Walker, Torsten Houwaart, Tobias Wienemann, Malte Kohns Vasconcelos, Daniel Strelow, Björn-Erik Ole Jensen, Tina Senff, Lisanna Hülse, Ortwin Adams, Marcel Andree, Sandra Hauka, Torsten Feldt, Verena Keitel, Detlef Kindgen-Milles, Jörg Timm, Klaus Pfeffer, Alexander T Dilthey, Catherine Moore, Aykut Ozdarendeli, Shaikh Terkis Islam Pavel, Hazel Yetiskin, Gunsu Aydin, Can Holyavkin, Muhammet Ali Uygut, Ceren Cevik, Alexey Shchetinin, Vladimir Gushchin, Gizem Dinler-Doganay, Levent Doganay, Tugba Kizilboga-Akgun, Ilker Karacan, Katarzyna Pancer, Piet Maes, Joan Martí-Carreras, Tony Wawina-Bokalanga, Bert Vanmechelen, Andrea Thürmer, Marianne Wedde, Ralf Dürrwald, Max Von Kleist, Oliver Drechsel, Thorsten Wolff, Stephan Fuchs, Rene Kmiecinski, Janine Michel, Andreas Nitsche, Inmaculada Casas, María Iglesias Caballero, Ángel Zaballos, Pilar Jiménez, Mercedes Jiménez, Sara Monzón Fernández, Sarai Varona Fernández, Isabel Cuesta De La Plaza, Artem Fadeev, Anna Ivanova, Mariia Sergeeva, Paola Stefanelli, M Estee Torok, Grant Hall, Ana da Silva Filipe, Lance Turtle, Safiah Afifi, Kathryn Mccluggage, Robert Beer, Juan Ledesma, Joshua Maksimovic, Karla Spellman, William L Hamilton, Angela Marchbank, Joel Alexander Southgate, Anthony Underwood, Ben Taylor, Corin Yeats, Khalil Abudahab, Matthew R Gemmell, Richard Eccles, Anita Lucaci, Charlotte Abigail Nelson, Lucille Rainbow, Mark Whitehead, Richard Gregory, Sam Haldenby, Steve Paterson, Margaret A Hughes, Martin D Curran, David Baker, Rachel Tucker, Luke R Green, Theresa Feltwell, Fenella D Halstead, Matthew Wyles, Aminu S Jahun, Shazaad S Y Ahmad, Iliana Georgana, Ian Goodfellow, Anna Yakovleva, Luke W Meredith, Artemis Gavriil, Ali Raza Awan, Chloe Fisher, Jonathan Edgeworth, Jessica Lynch, Nathan Moore, Rebecca Williams, Stephen P Kidd, Nicholas Cortes, Kirstyn Brunker, John T Mccrone, Joshua Quick, Nichola Duckworth, Sarah Walsh, Tim Sloan, Catherine Ludden, Ryan P George, Gary Eltringham, Julianne R Brown, Elihu Aranday-Cortes, James G Shepherd, Joseph Hughes, Kathy K Li, Thomas C Williams, Natasha Johnson, Natasha Jesudason, Daniel Mair, Emma Thomson, Rajiv Shah, Yasmin A Parr, Stephen Carmichael, David L Robertson, Kyriaki Nomikou, Alice Broos, Marc Niebel, Katherine Smollett, Lily Tong, Shahjahan Miah, Anita Wittner, Nicole Phillips, Brendan Payne, Rebecca Dewar, Alison Holmes, Frances Bolt, James R Price, Siddharth Mookerjee, Dheeraj K Sethi, Will Potter, Rachael Stanley, Reenesh Prakash, Samir Dervisevic, Jonathan Clive Graham, Andrew Nelson, Darren Smith, Gregory R Young, Wen Chyin Yew, John A Todd, Amy Trebes, Monique Andersson, Matthew Bull, Joanne Watkins, Alec Birchley, Bree Gatica-Wilcox, Lauren Gilbert, Sara Kumžiene-Summerhayes, Sara Rey, Anoop Chauhan, Ethan Butcher, Kelly Bicknell, Scott Elliott, Sharon Glaysher, Angie Lackenby, David Bibby, Steven Platt, Hodan Mohamed, Nicholas William Machin, Jean Lutamyo Mbisa, Jonathan Evans, Malorie Perry, Nicole Pacchiarini, Sally Corden, Alexander Geraint Adams, Amy Gaskin, Jason Coombs, Lee John Graham, Simon Cottrell, Mari Morgan, Laura Gifford, Anastasia Kolyva, Steven John Rudder, Alexander J Trotter, Alison E Mather, Alp Aydin, Andrew J Page, Gemma L Kay, Leonardo de Oliveira Martins, Muhammad Yasir, Nabil-Fareed Alikhan, Nicholas M Thomson, Rachel Gilroy, Robert A Kingsley, Justin O'Grady, Ana Victoria Gutierrez, Maria Diaz, Thanh Le Viet, Ana P Tedim, Evelien M Adriaenssens, C Patrick Mcclure, Christopher Moore, Fei Sang, Gemma Clark, Hannah C Howson-Wells, Johnny Debebe, Jonathan Ball, Joseph Chappell, Manjinder Khakh, Matthew Carlile, Matthew Loose, Michelle M Lister, Nadine Holmes, Theocharis Tsoleridis, Vicki M Fleming, Victoria Wright, Wendy Smith, Michael D Gallagher, Matthew Parker, David G Partridge, Cariad Evans, Paul Baker, Sarah Essex, Steven Liggett, Alexander J Keeley, Matthew Bashton, Stefan Rooke, Samir Dervisevic, Emma Jane Meader, Carlos Enrique Balcazar Lopez, Adrienn Angyal, Mark Kristiansen, Helena J Tutill, Jacqueline Findlay, Lamia Mestek-Boukhibar, Leysa Forrest, Patricia Dyal, Rachel J Williams, Yasmin Panchbhaya, Charlotte A Williams, Sunando Roy, Sarojini Pandey, Jo Stockton, Nicholas J Loman, Radoslaw Poplawski, Samuel Nicholls, W P M Rowe, Fahad Khokhar, Malte Lars Pinckert, Myra Hosmillo, Yasmin Chaudhry, Laura G Caller, Rose K Davidson, Luke Griffith, Andrew Rambaut, Ben Jackson, Rachel Colquhoun, Verity Hill, Jenna Nichols, Patawee Asamaphan, Alistair Darby, Kathryn A Jackson, Miren Iturriza-Gomara, Ecaterina Edith Vamos, Angie Green, David Aanensen, David Bonsall, David Buck, George Macintyre-Cockett, Mariateresa de Cesare, Oliver Pybus, Tanya Golubchik, Garry Scarlett, Katie F Loveson, Samuel C Robson, Angela Beckett, Benjamin Lindsey, Danielle C Groves, Paul J Parsons, Martin P Mchugh, James Daniel Barnes, Carmen F Manso, Dimitris Grammatopoulos, Katja Elisabeth Menger, Ewan Harrison, Rory Gunson, Sharon J Peacock, Gabriel Gonzalez, Michael Carr, Lazar Mihaela, Odette Popovici, Mia Brytting, Catherine Bresner, William Fuller, Trudy Workman, Andreas F Mentis, Athanasios Kossyvakis, Timokratis Karamitros, Vasiliki Pogka, Antonios Kalliaropoulos, Elina Horefti, Aspasia Kontou, Beatriz Martinez-Gonzalez, Voula Labropoulou, Androniki Voulgari-Kokota, Maria Evangelidou, Panagiota Bizta, Maria Belimezi, Laurens Lambrechts, Mehmet Z Doymaz, Merve Kalkan Yazici, Nesibe S Cetin, Elif Karaaslan, Hannimari Kallio-Kokko, Jenni Virtanen, Maija Suvanto, Phuoc Truong Nguyen, Pekka Ellonen, Sari Hannula, Harri Kangas, Vattipally B Sreenu, Katalin Burián, Gabriella Terhes, Katalin Gombos, Attila Gyenesei, Péter Urbán, Róbert Herczeg, Ferenc Jakab, Gábor Kemenesi, Gábor Endre Tóth, Balázs Somogyi, Brigitta Zana, Safia Zeghbib, Anett Kuczmog, Fanni Földes, Zsófia Lanszki, Mónika Madai, Henrietta Papp, Ágnes Nagy, Csaba István Pereszlényi, Gergely Csaba Babinszky, Gábor Dudás, Eszter Csoma, Ahmad N Abou Tayoun, Alawi A Alsheikh-Ali, Tom Loney, Norbert Nowotny, Osama Abdul-Wahab, Fernando Gonzalez-Candelas, Martin H Andersen, Sarah Taylor

**Affiliations:** 1 The European Centre for Disease Prevention and Control, Stockholm, Sweden; 2 Public Health Wales Microbiology, Cardiff, United Kingdom; 3 Cardiff University School of Biosciences, Cardiff, United Kingdom; 4 Biozentrum, University of Basel, Basel, Switzerland; 5 Smorodintsev Research Institute of Influenza, Saint Petersburg, Russian Federation; 6 Bioinformatics Institute, Agency for Science Technology and Research, Singapore, Singapore; 7 Department of Biological Sciences, National University of Singapore, Singapore, Singapore; 8 National Public Health Laboratory, National Centre for Infectious Diseases, Ministry of Health, Singapore, Singapore; 9 Global Initiative on Sharing All Influenza Data (GISAID), Munich, Germany; 10 University of Edinburgh, Edinburgh, United Kingdom; 11 Health Emergency Programme, WHO Regional Office for Europe, Copenhagen, Denmark; 12 The members of the working group are listed in the Investigator tab

**Keywords:** COVID-19, SARS-CoV-2, NGS, WGS, sequencing, nomenclature, Europe

## Abstract

We show the distribution of severe acute respiratory syndrome coronavirus-2 (SARS-CoV-2) genetic clades over time and between countries and outline potential genomic surveillance objectives. We applied three genomic nomenclature systems to all sequence data from the World Health Organization European Region available until 10 July 2020. We highlight the importance of real-time sequencing and data dissemination in a pandemic situation, compare the nomenclatures and lay a foundation for future European genomic surveillance of SARS-CoV-2.

During the coronavirus disease (COVID-19) pandemic, whole genome sequencing (WGS) has been used extensively by laboratories all over the world to characterise the virus. To be able to implement continuous monitoring of genetic changes through WGS as part of surveillance at the European level, surveillance objectives and virus nomenclatures need to be defined. In this report, we applied the available nomenclatures to the European subset of the GISAID dataset to describe broad geographical and temporal trends in the distribution of SARS-CoV-2 genetic clades during the first half of 2020 and we discuss potential genomic surveillance objectives at the European level.

## Genome data availability during the COVID-19 pandemic

The COVID-19 pandemic is the first pandemic where WGS capacity has been widely available to the public health sector from the beginning. Since the first genomes were published in January 2020 [1-4], the number of available sequences has rapidly increased to more than 63,000 complete genome sequences available in GISAID as at 10 July [5]. The World Health Organization (WHO) European Region has been an active contributor, with around 39,000 complete genome sequences from 35 countries published in GISAID, with a median time of 40 days from sample collection to publication according to the GISAID collection and submission dates. Of those sequences, 8% were published within 2 weeks of collection and 0.6% of sequences were published within 1 week of collection. Supplementary Figure S1 summarises the number of genomes sampled and submitted per month. A detailed account of the turnaround time for each country is shown in Supplementary Figure S2. This demonstrates that with enough resources, it is possible to set up and perform real-time WGS and global analysis of a newly emerged virus.

## Clade nomenclatures for SARS-CoV-2

One major component of sequence-based surveillance of any pathogen is applying meaningful nomenclatures to the sequence data, based on the genetic relatedness of the sequences. This streamlines communication between different actors in the molecular epidemiology field and enables simplified tabulation of the genomic data for integration with classical epidemiological analysis.

**Figure 1:**
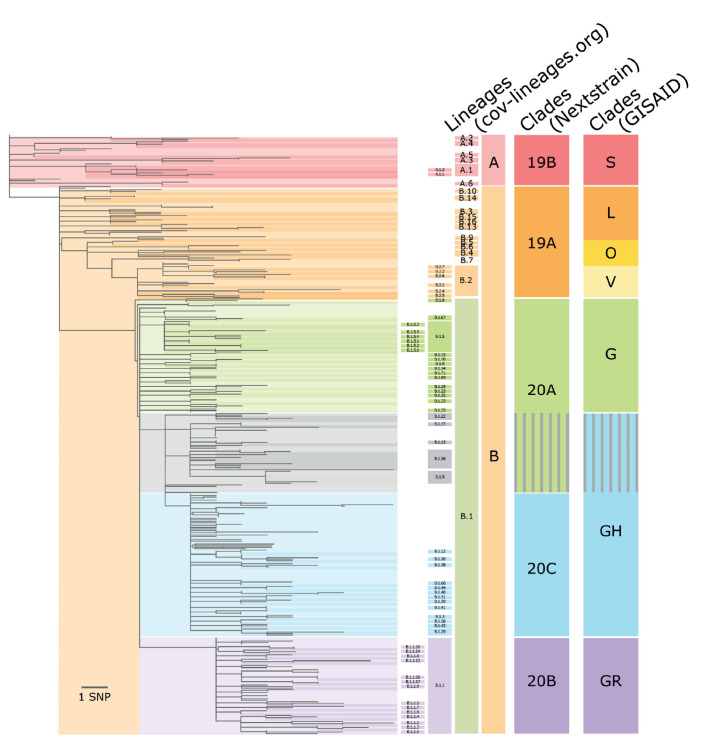
Schematic comparison of the GISAID, Nextstrain and cov-lineages.org nomenclatures for SARS-CoV-2 sequences of world-wide origin, February–July 2020

Several nomenclatures have been introduced for SARS-CoV-2 (strains in GISAID use the name hCoV-19), including by Nextstrain [6], GISAID [7] and Rambaut et al. (cov-lineages.org) [8-10]. While Nextstrain and GISAID clade nomenclatures aim at providing a broad-brush categorisation of globally circulating diversity, the lineages (cov-lineages.org) are meant to correspond to outbreaks. The relation between the three nomenclatures is illustrated in Figure 1.

SNP: single nucleotide polymorphism.

A schematic tree is shown, representing the major topological features of the SARS-CoV-2 phylogeny. The tree is shaded to correspond to the major lineages and all Nextstrain and GISAID clades. Between Nextstrain clades 20A and 20C and GISAID clades G and GH, there are differences in the position where clades are divided (shown as grey in the main tree and lineage columns, and grey stripes in the Nextstrain and GISAID column). Here, the grey is shown as stripes in the Nextstrain and GISAID clade columns to reflect how they are categorised by each methodology. Association between the lineages and clades is approximate. The sequence data available from GISAID were curated using the Pangolin data preparation pipeline [8]. In brief, we masked out singleton substitutions identified in the pipeline and selected a set of representatives per lineage or clade. We aligned the sequences using MAFFT v7.470 [14] and estimated a maximum likelihood phylogeny using IQ-Tree v1.6.12 with 10,000 ultrafast bootstraps [15,16]. Sequence EPI_ISL_406801 (Wuhan/WH04/2020), a basal A lineage sequence, was used as an outgroup for the tree. We visualised the tree using baltic with custom python scripts and manual edits [17].

Agreement on a single nomenclature is a major global undertaking that cannot be decided at the national or European level. Major success factors of nomenclature systems include adoption of the nomenclature by major actors in the field, fitness for purpose to the surveillance objectives, compliance with the General Data Protection Regulation (GDPR), and availability on software platforms. Currently, the available SARS-CoV-2 nomenclatures do not attempt to reflect any antigenic or other phenotypic properties of the virus.

## Distribution of clades over time

Sequence data were downloaded from GISAID on 10 July 2020.

**Figure 2:**
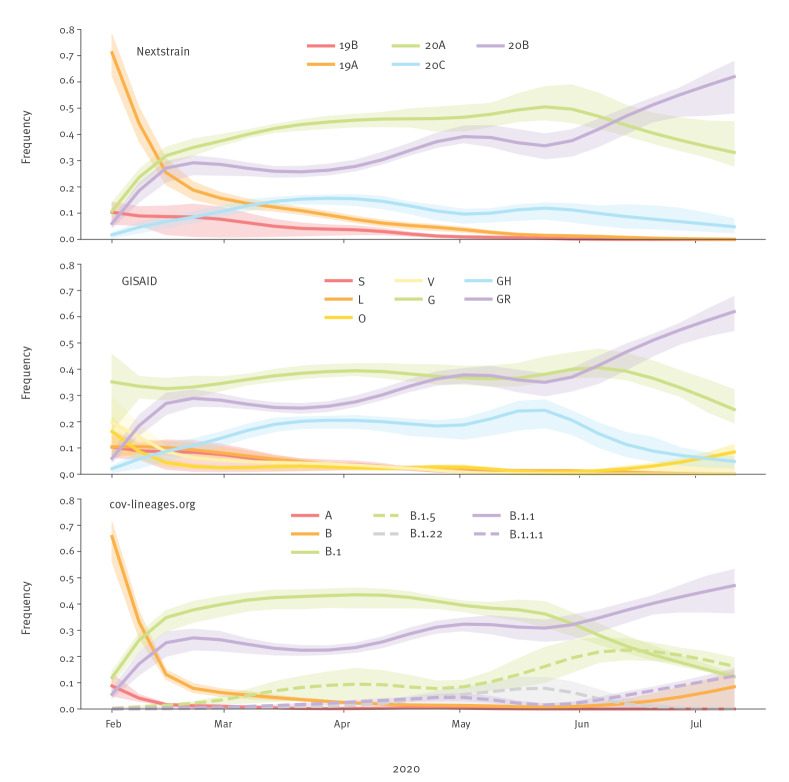
Frequency trajectories of SARS-CoV-2 clades and lineages by collection date, WHO European Region, February–July 2020 (n = 37,187, subsampled to 3,324)

The subsampled distribution of clades and lineages over time in the WHO European Region is shown in Figure 2. There was an initial period in January 2020 when the 19A/L/V/O clades (Nextstrain/GISAID nomenclatures) were more prevalent in total than the 20A/G clades, However, this could partially be an effect of small numbers of sequenced viral genomes in Europe early in the pandemic as well as the widespread sampling strategy focusing on cases with travel history to East Asia. The 20A/G clades are characterised by the spike protein D614G mutation that has been suggested to increase transmissibility but not pathogenicity [11]. After this initial stage, the 20B/GR clade increased rapidly, stabilised around 30% between March and May 2020 and increased further to become the most frequent clade in June 2020. The trajectory for the 20C/GH clades differs slightly depending on the nomenclature applied. The Nextstrain 20C clade peaked at ca 20% of the sequences in early April 2020 and has since then slowly declined and almost disappeared, while the GISAID GH clade peaked at ca 30% in May 2020 and has rapidly declined since then. The changing trends at the end of the data series could partially be explained by different delays from sampling to publication for different countries with different clade distributions.

From top to bottom: Nextstrain, GISAID and cov-lineages.org. Shaded areas: interquartile ranges obtained by bootstrap resampling the dataset by country. Fewer sequences from fewer countries are available in recent weeks, resulting in widening confidence intervals. Frequency trajectories were generated from a sample representative across sampling time and space using the augur filter routine and selecting 15 random sequences for each month and administrative division [18]. When fewer than 15 sequences were available, all data were included. Frequencies were calculated using Gaussian smoothing with a standard deviation of 10 days. Script is available from https://github.com/neherlab/ncov-ecdc/.

When applying the cov-lineages.org nomenclature, the main A and B lineages were prevalent in January and then diversified rapidly into sublineages which had stable frequencies from late February 2020 to end of April 2020; thereafter, an increase in the frequency of B.1.1 (corresponds to clade 20B/GR) and B.1.5 (part of clade 20A/G) lineages can be observed.

## Clade distribution in the WHO European Region countries

**Figure 3:**
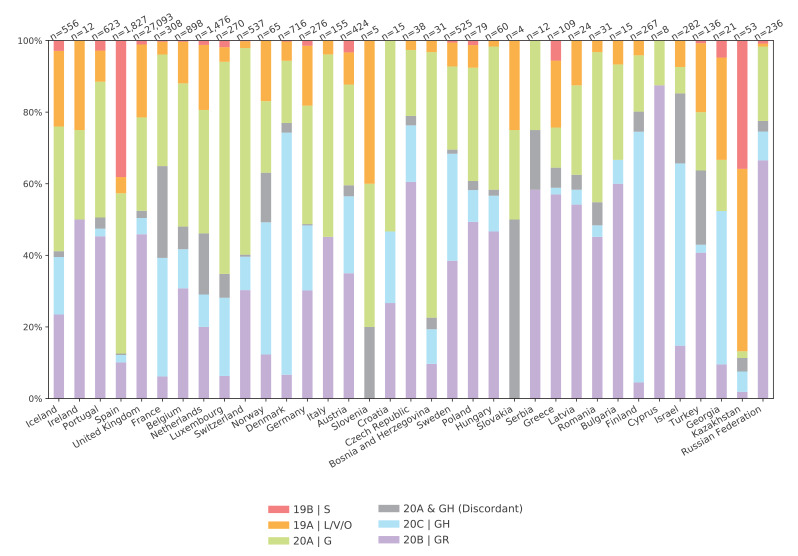
Distribution of GISAID and Nextstrain clades of SARS-CoV-2 across countries in the WHO European Region, based on all high-quality genomes in GISAID, February– July 2020 (n = 37,187)

The geographical distribution of Nextstrain and GISAID clades shows that in general, clade 19B/S has been very rare, except in Spain and Kazakhstan, and frequencies of the other clades were varied for most countries in the WHO European Region (Figure 3). For countries with few sequences, the clade distribution may be unreliable, and there may be a bias towards the clades that dominated at different stages of the pandemic depending on varying sampling strategies over time and the timeliness of the submission of sequences.

The numbers at the top of each bar indicate the number of available genomes. The grey areas represent sequences called differently by the GISAID and Nextstrain methodologies, and correspond to the grey-shaded partition of the tree in Figure 1. Supplementary Figure S3 shows the same data using the cov.lineages.org nomenclature. The proportion of clades found in each country was calculated using all available full-length, high-quality (> 27,000 bases, < 3,000 undetermined bases) sequences available on GISAID. Clade membership for each sequence was determined by given GISAID annotation or via the Nextstrain clades script, and proportions were calculated for all sequences from a given country. Script is available from https://github.com/neherlab/ncov-ecdc/

Among the countries with more than 100 sequences available, Spain stands out with a high proportion of clades 19B/S and 20A/G and very little of the other clades, Denmark, Finland and Israel with a high proportion of 20C/GH, Greece, Italy, Portugal and the United Kingdom with no or very little 20C/GH and the Russian Federation and Switzerland with very little 19A/L/V/O. The distribution may depend on founder effects and sampling bias, but also on when and for how long travel restrictions and national response measures were implemented. Early travel restrictions would probably have reduced the early incidence and therefore the 19A/L/V/O clades that were most prevalent before the 20A/G-clades became dominant, while travel restrictions and lockdowns implemented later could have preserved the clade distribution as it was at the time of the implementation.

## Discussion about SARS-CoV-2 genomic surveillance in the WHO European Region

Through discussions in a call open to all laboratories in the WHO European region submitting SARS-CoV-2 sequences to GISAID, we identified the following key surveillance objectives for the use of SARS-CoV-2 sequence data at the European level.

Current (January 2020 and onward):

- Investigating transmission dynamics and introductions of novel genetic variants;
- Investigating the relationship between clades/lineages and epidemiological data such as transmissibility and disease severity or risk groups to guide public health action;
- Understanding the impact of response measures on the virus population;
- Assessing the impact of mutations on the performance of molecular diagnostic methods;
- Assessing the impact of mutations on the performance of serological methods.

Future (when antiviral drugs and vaccines become available):

- Assessing the impact of mutations on the performance of antiviral drugs;
- Assessing the impact of mutations and modelling the antigenic properties of the virus to assess the risk of vaccine escape.

All of these objectives benefit from a representative sample of the virus population. The first three also depend to a large degree on data completeness, which is a limitation in the currently available dataset where the number of sequences per country ranges from four to 28,000. Rapid sequencing is crucial for real-time outbreak analysis, for instance to show that the flare-up in June 2020 in Beijing [12] was caused by virus variants similar to those circulating in Europe or to demonstrate separate introductions to mink farms in the Netherlands [13].

The experience of sequencing SARS-CoV-2 in Europe to date demonstrates the potential for rapidly sharing genomic data to support national and international public health response. A genomic surveillance guidance that takes the variable resources in the region into account is necessary to fully realise this potential. GISAID made a global, curated sharing system for SARS-CoV-2 sequences available early in the pandemic, and the benefits of available tools for early sharing of sequences have been demonstrated during this pandemic through rapid implementation of diagnostic tests and monitoring of the viral characteristics over time. Implementing real-time genomic surveillance at the European level could further elucidate differences in circulating strains between countries and enhance the understanding of how response measures, and later vaccines and antiviral drugs, affect the proportion of genetic variants. 

## Conclusions

Overall, the GISAID and Nextstrain nomenclatures provide similar pictures of the situation and may provide useful systems for genomic situation reporting globally. The cov-lineages.org nomenclature provides information at a finer scale and has the potential to provide early warning of expanding lineages that may represent regional outbreaks or later become dominant because of some selective advantage such as vaccine escape or increased transmissibility.

## Supplementary Data

952000 Supplement
