# Authors’ correction for Euro Surveill. 2020;25(32)

**DOI:** 10.2807/1560-7917.ES.2020.25.33.200820c

**Published:** 2020-08-20

**Authors:** Eurosurveillance editorial team

**Affiliations:** 1 European Centre for Disease Prevention and Control (ECDC), Stockholm, Sweden

In the article ‘Geographical and temporal distribution of SARS-CoV-2 clades in the WHO European Region, January to June 2020’ by Alm et al. published on 13 August 2020, the name of Paula Ruiz-Rodriguez was left out in the list of members of the WHO European Region sequencing laboratories and GISAID EpiCoV group. On request of the authors, this author was added to the group on 20 August 2020.

