# Supplementary material for: Geographical and temporal distribution of SARS-CoV-2 clades in the WHO European Region, January to June 2020
Source: Euro Surveill. 2020 Aug 13;25(32):2001410. doi: 10.2807/1560-7917.ES.2020.25.32.2001410 (PMC7427299; doi:10.2807/1560-7917.ES.2020.25.32.2001410)

Supplementary material

This supplementary material is hosted by Eurosurveillance as supporting information alongside the article Geographical and temporal distribution of SARS-CoV-2 clades in the WHO European Region, January to June 2020, on behalf of the authors, who remain responsible for the accuracy and appropriateness of the content. The same standards for ethics, copyright, attributions and permissions as for the article apply. Supplements are not edited by Eurosurveillance and the journal is not responsible for the maintenance of any links or email addresses provided therein.

Figure S1. Distribution of sample collection and sequence submission dates of all sequences in GISAID from the WHO European Region until 10 July 2020.

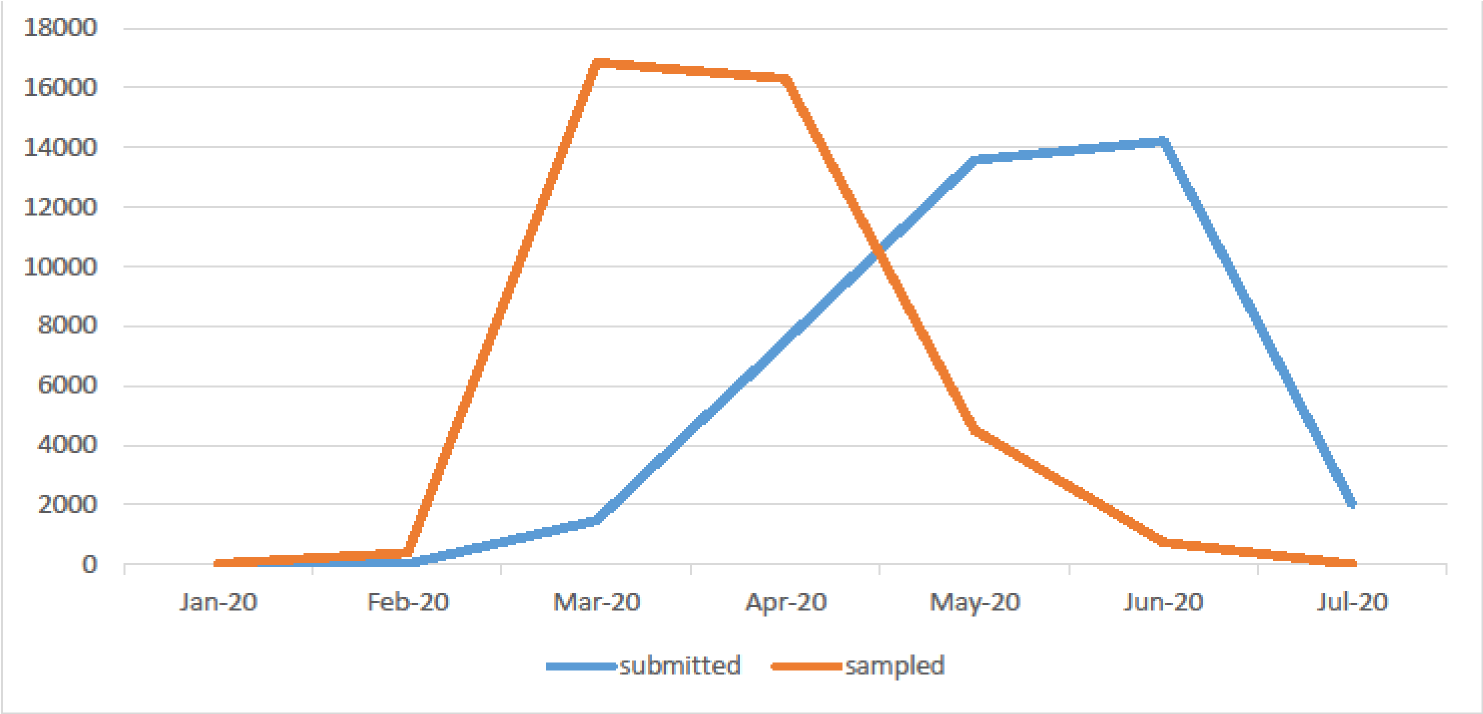

Figure S2. Turnaround time between sample collection and sequence submission for all countries in the WHO European Region that have submitted sequences to GISAID EpiCoV until 10 July 2020. The green figures indicate the mean turnaround time and the red figures indicate the number of genomes submitted.

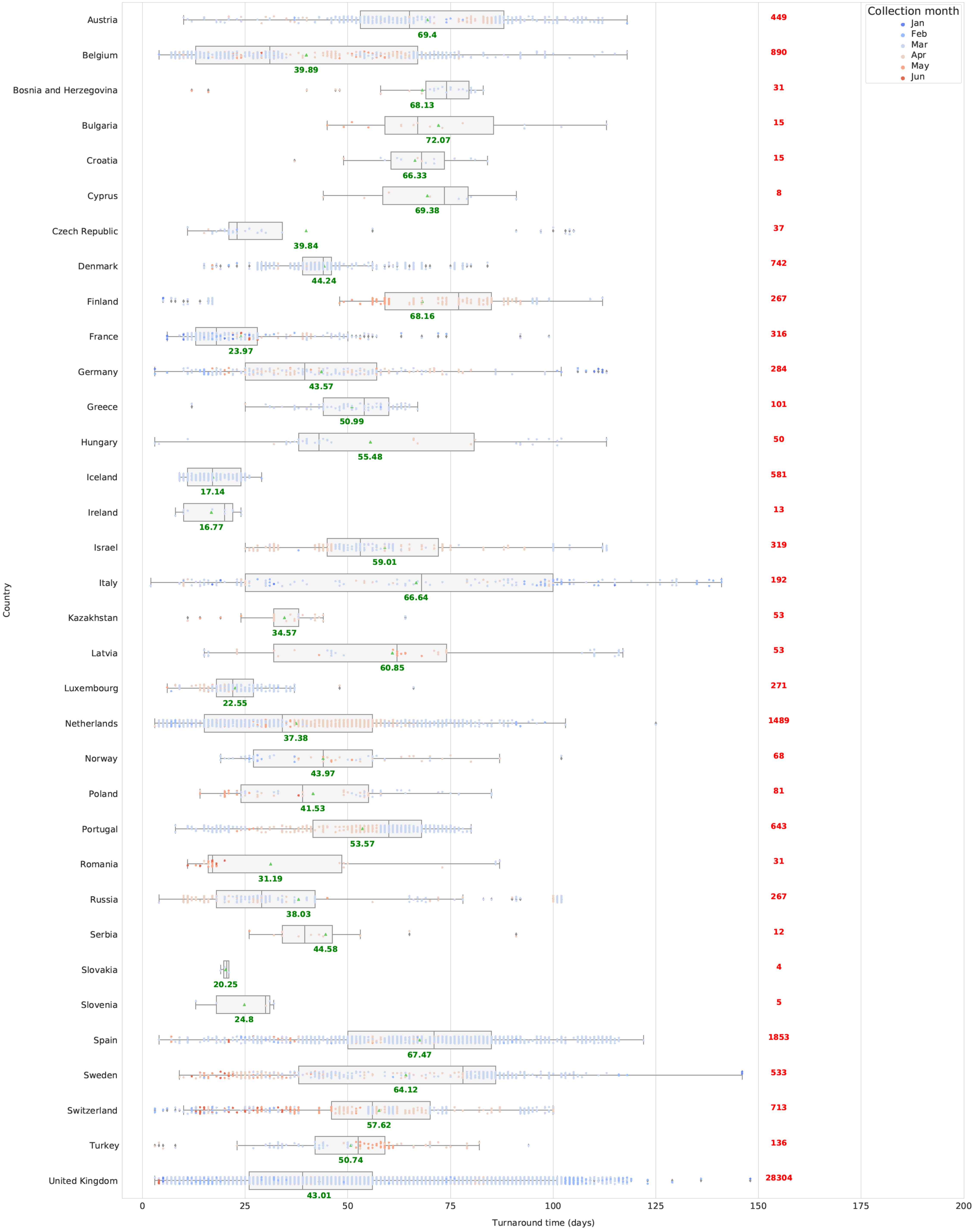

Figure S3. Distribution of the most common lineages (cov-lineages.org) in the WHO European Region, based on all high-quality genomes in GISAID until 10 July 2020.

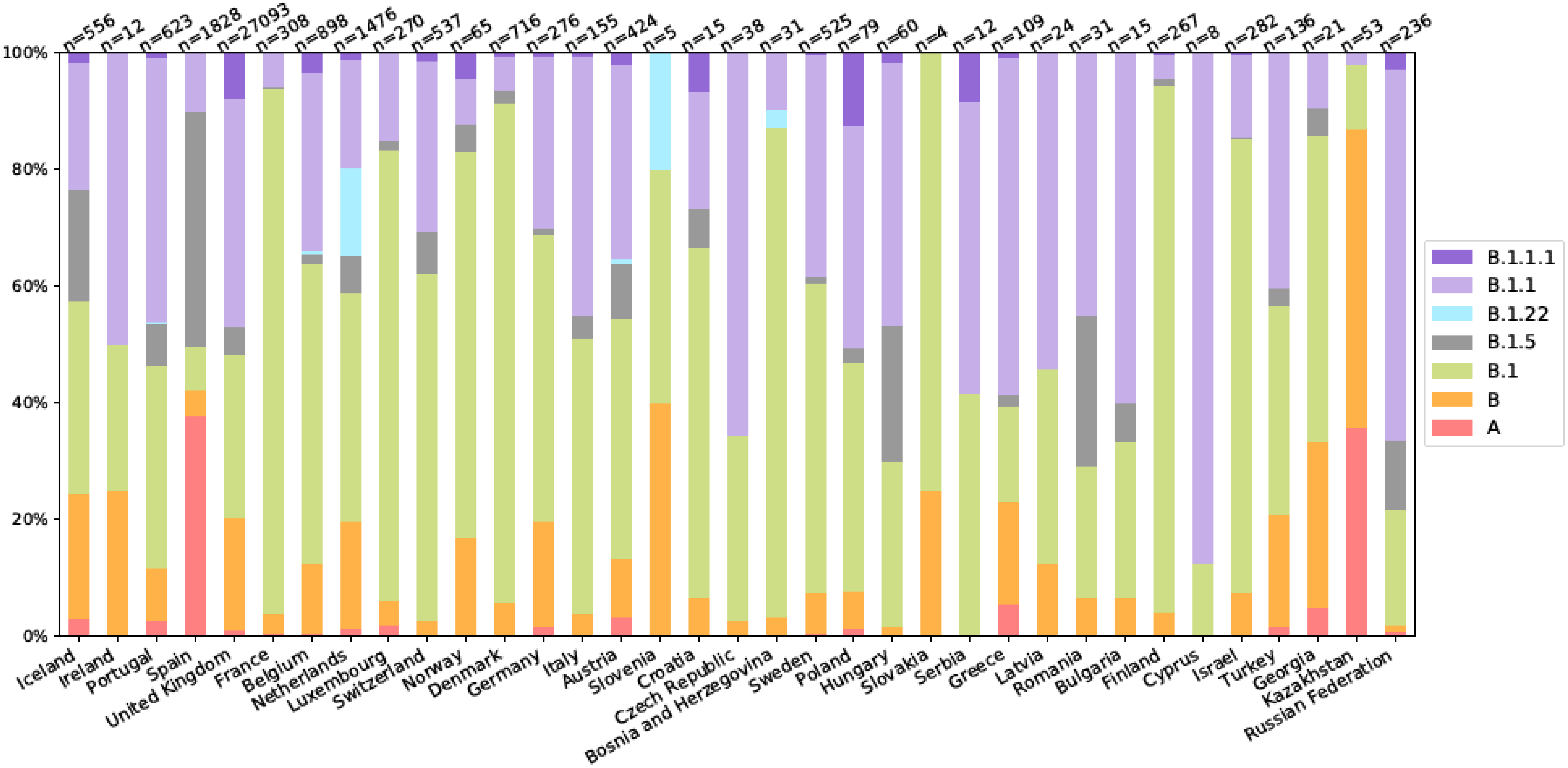

Supplement: Supplement [file 20-01410_Supplement_ALM.pdf]
